# The effect of applying ethnicity-specific spirometric reference equations to Asian migrant workers in Korea

**DOI:** 10.1186/s40557-015-0065-0

**Published:** 2015-05-18

**Authors:** Nami Kim, Se-Yeong Kim, Yoojun Song, Chunhui Suh, Kun-Hyung Kim, Jeong-Ho Kim, Byung-Chul Son, Chae-Kwan Lee, Jong-Tae Lee

**Affiliations:** Na-Eun Hospital, Gajwa-3dong 277-7, 8, Seo-gu, Incheon, Republic of Korea; Department of Occupational and Environmental Medicine & Institute of Environmental and Occupational Medicine, Inje University Busan Paik Hospital, Bokji-ro 75, Busanjin-gu, Busan, Republic of Korea

**Keywords:** Spirometry, Ethnicity, Reference equations

## Abstract

**Objectives:**

Asian migrant workers in Korea have various ethnicities. The purpose of this study was to evaluate the difference in spirometric interpretation made using the set of third National Health and Nutrition Examination Survey (NHANES III) reference equations and the relevant ethnicity-specific reference sets.

**Methods:**

Spirometry was performed on 166 migrant and 498 Korean male workers between March and November 2012. We analyzed the spirometric data of healthy never-smokers. Spirometric patterns were evaluated using the NHANES III reference set and some relevant ethnicity-specific equations (Eom’s equation for Koreans, Ip’s equation for East Asians, Crapo’s equation for Central Asians, Memon’s equation for South Asians, and Gnanou’s equation for Southeast Asian people).

**Results:**

In all migrant groups except the Central Asian group, the forced expiratory volume in 1 second percentage (FEV_1_%) and forced vital capacity percentage (FVC%) calculated using each of the ethnicity-specific reference equations considered were significantly higher than those calculated using the NHANES III reference set. This study showed that in the evaluation of the spirometric result of subjects from Southeast Asia or South Asia, the percentage of cases with an abnormal FEV_1_ or FVC increased when the NHANES III set of equations was used as compared to when the ethnicity-specific equations were used.

**Conclusions:**

We found that the spirometric results of all ethnic groups were higher and the composition of the abnormal spirometric result was lower when the ethnicity-specific reference equations were used instead of the NHANES III reference set.

## Introduction

The Occupational Safety and Health Act in Korea requires that a spirometric test be done as a regular health checkup for employees exposed to pulmonary hazards in the workplace, such as dust, metal fumes, oil mist, or organic solvents that can lead to some ventilatory disorders like occupational asthma, chronic obstructive pulmonary disease (COPD), and interstitial lung disease. Some parameters measured during a spirometric test include forced vital capacity (FVC), forced expiratory volume in 1 second (FEV_1_), FEV_1_/FVC, and peak expiratory flow (PEF) [1]. A spirometric test is an effective and reliable diagnostic tool for assessing obstructive ventilatory disorders such as asthma or COPD by using FEV_1_/FVC and PEF, and restrictive ventilator disorders such as lung parenchymal disease by using FVC. For the interpretation of a spirometric test, a normal pulmonary function reference value is necessary for determining the normal range, and this value is calculated using spirometric reference equations. The job fitness of employees and work-relatedness of reduced pulmonary function are judged with a simultaneous consideration of the spirometric result, work-related symptoms, and workplace respiratory hazards [2,3].

The number of migrant workers in Korea has increased. In 2012, 1,445,103 non-Korean citizens were employed in Korea and 230,237 of them had an E-9 visa for non-professional work [4]. China and Southeast Asia are the most common regions of origin of the migrant laborers in Korea and account for 79% of the non-Koreans in Korea. Further, since many immigrants, mainly from Southeast Asia, remain undocumented, the actually ratio of migrant workers from Asian countries is known to be higher than the calculated value [5]. Considering the diversity of language families in Asia, it is supposed that Asians have a number of ethnic origins [6].

Respiratory impairment is influenced by ethnic differences [7]. The variation of ethnic spirometric reference equations has been previously reported in the literature [8-10], and official recommendations have been made. The most recent guidelines, published in 2005 by the American Thoracic Society (ATS)/European Respiratory Society (ERS), recommend ethnicity-specific reference standards. The ERS/Global Lung Initiative task force announced spirometric reference equations (ERS/GLI 2012) derived from data collected from healthy nonsmokers in the age group of 3–95 years from 33 countries. The ERS/GLI 2012 equations provided multi-ethnic values and the lower limit of normal (LLN) for spirometry [11,12]. However, many countries still have no choice but to use pulmonary function reference equations that have not been developed for the local ethnic groups. Most spirometric tests use European descent as the base population for a prediction equation of normal reference values [13]. Therefore, a spirometric interpretation, that is, a spirometric reference equation developed considering the ethnicity of subjects, is needed [14,15]. Spirometric values of Asians in general are known to be lower than those of Caucasians, but spirometric values of Koreans in particular are not lower than those of Caucasians [16-19].

In this study, we compared the spirometric results of migrant workers and Korean workers in Busan metropolitan city and compared the interpretations of the spirometric data determined by ethnicity-specific reference equations with those determined by the third National Health and Nutrition Examination Survey (NHANES III) reference equation set.

## Materials and methods

### Subjects

The subjects in this study were 3,067 male workers who underwent spirometry as part of a regular health examination in the occupational health examination center of a hospital located in Busan between March and November 2012. All subjects were Korean and non-ethnic-Korean migrant workers. In this study, we considered 1658 subjects in the age group of 20 to 40 years because most migrant workers belong to this age group. We excluded 24 subjects (22 Korean workers and 2 migrant workers) with an abnormal chest radiograph or unconfirmed nationality, or with a history of significant lung or heart disease, or with respiratory symptom such as persistent cough, persistent phlegm production, etc. Subjects with a satisfactory result with respect to suitability and reproducibility were selected for inclusion by analyzing the spirometry data using the technical guidelines of the spirometric test and interpretation of the Korea Occupational Safety and Health Agency [2]. 166 migrant workers and 1,434 Korean workers showed satisfactory results. Three Korean subjects were matched with each migrant subject randomly selected with respect to age and smoking history. Finally, a total of 498 Korean and 166 migrant workers were enrolled.

### Ethnicity-specific spirometric equations

The migrant workers were classified into the following four groups on the basis of the subjects’ country of origin: East Asia, Southeast Asia, Central Asia, and South Asia. East Asia consisted of China, Japan, and Taiwan. Southeast Asia consisted of Brunei, Cambodia, Indonesia, Laos, Malaysia, Myanmar, Philippines, Singapore, Thailand, and Vietnam. Central Asia included Kazakhstan, Kyrgyzstan, Tajikistan, Turkmenistan, Uzbekistan, and Mongolia. South Asia included Afghanistan, Bangladesh, Bhutan, India, Maldives, Nepal, Pakistan, and Sri Lanka.

We selected an ethnicity-specific reference equation for each of the abovementioned subregions on the basis of a literature review. We searched for studies of spirometric reference equations that had been established for each of the subregions and for Korea that met the following criteria: First, spirometry had been performed on the basis of the ATS guidelines. Second, spirometric equations for the FEV_1_ and FVC had been devised. Third, the studies needed to have been based on subjects belonging to the age group of 20 to 40 years. Fourth, the number of subjects was higher than 500. Fifth, the studies must have been published in recent years, at least in or after 1999. We could not find the equations developed for Central Asia that met the fourth criterion, and therefore, the selected study for Central Asia had 344 subjects. The selected spirometric reference equations of each subregion were as follows: Eom’s equation for Koreans, Ip’s equation from Hong Kong for East Asians, Gnanou’s equation from Malaysia for Southeast Asians, Crapo’s equation from Mongolia for Central Asians, and Memon’s equation from Pakistan for South Asians. Although the most limited definition of Central Asia does not include Mongolia, no other spirometric reference has been developed recently in Central Asia strictly defined. The former definition of Central Asia by United Nations Educational, Scientific, and Cultural Organization (UNESCO) included Mongolia [20]. The reference equation used for the group of Central Asians was developed in Mongolia [9,21-25] (Table 1).

**Table 1:** **Predicted FEV**_**1**_ **and FVC of a given 30-year-old, 70-kg, 170-cm-tall male calculated using spirometric reference equations**

**Spirometric equations**	**Country of development**	**Number of subjects**		**Predicted equations** ^*^		**Predicted value (L)**
		**Male**	**Female**			
NHANES III (1999)	USA	898	1383	FEV_1_	0.5536 – 0.01303a – 0.00017a^2^ + 0.000141h^2^	4.08
				FVC	−0.1933 + 0.00064a – 0.00027a^2^ + 0.000157h^2^	4.12
Ethnicity-specific equations						
Korean (2013)	South Korea	706	4047	FEV_1_	0.61493 – 0.00025410a^2^ + 0.00012644h^2^ – 0.00262w	3.30
				FVC	1.19135 – 0.00000219a^3^ + 0.0000006995642h^3^	4.57
East Asian (2006)	Hong Kong, China	494	595	FEV_1_	2.404 – 0.0254a + 0.03978h	3.60
				FVC	4.424 – 0.0193a + 0.05434h	4.23
Southeast Asian (2011)	Malaysia	222	310	FEV_1_	– 1.284 – 0.027a + 0.03167h	3.29
				FVC	2.176 – 0.027a + 0.03889h	3.63
Central Asian (1999)	Mongolia	176	168	FEV_1_	2.0956 – 0.02411a + 0.04052h	4.07
				FVC	3.5922 – 0.01532a + 0.05207h	4.80
South Asian (2007)	Pakistan	321	183	FEV_1_	1.440 – 0.020a + 0.030h	3.06
				FVC	0.848 – 0.020a + 0.032h	3.99

*a: age (year), h: height (cm), w: weight (kg).

FEV; Forced expiratory volume, FVC; Forced vital capacity, NHANES; National Health and Nutrition Examination Survey.

### Data collection

The subjects’ basic data such as nationality, workplace, job position, smoking history, age, height, and weight were collected. A chest radiograph was performed. The participants were asked about their smoking history through a self-administered questionnaire before the spirometry.

For the spirometry, FVC and FEV_1_ were measured. Spirometry was performed in subjects in stable condition after explaining its purpose and the required position. The spirometry was performed with the examinee in a seated position after one practice test. The better result of three attempts was selected. A FlowScreen testing device (Cardinal Health, Inc., Germany) was used for the screening. Standardized pulmonary function tests were performed according to the ATS/ERS guidelines [11].

Each migrant worker’s country of origin was stratified into four groups: East Asia, Southeast Asia, Central Asia, and South Asia. By applying the NHANES III reference set, we compared the spirometric results of each group with those of the Korean workers. Then, the results of applying ethnicity-specific spirometric references were compared with those of applying the NHANES III reference set. To acquire values with percentages, the actual measured FEV_1_ and FVC were divided into the calculated FEV_1_ and FVC from each equation (FEV_1_% = measured FEV_1_/calculated FEV_1_, FVC% = measured FVC/calculated FVC). Abnormal FVC and FEV_1_ values were defined as values that were less than 80% of the reference value [26]. Subjects were divided into three groups according to their smoking status: never smoker (n = 183 for Korean workers, n = 88 for all migrant workers), ex-smoker (n = 56 for Korean workers, n = 11 for all migrant workers), and current smokers at the time the spirometry tests were conducted (n = 259 for Korean workers, n = 67 for all migrant workers). As cigarette smoke affects lung function and may be considered a potentially confounding factor, current and ex-smokers were excluded from the statistical analysis.

### Statistical analysis

The differences in the values of the FEV_1_% and FVC% between results obtained by applying NHANES III and the ethnicity-specific equations for the never smokers were analyzed using a paired *t*-test. The differences in the values of the FEV_1_% and FVC% between never-smoker Korean and migrant workers obtained by applying the NHANES III set of equations and the ethnicity-specific sets were analyzed using a *t*-test. The differences in the frequency of abnormal FEV_1_ and FVC were analyzed with McNemar’s test. The evaluation agreement for abnormal FVC and FEV_1_ obtained using the reference equations and the concordance coefficients were analyzed using the Kappa distribution. IBM SPSS Statistics version 21.0 (IBM, Corp., Armonk, NY, USA) was used for the analysis.

## Results

### Subjects’ characteristics

**Figure 1:**
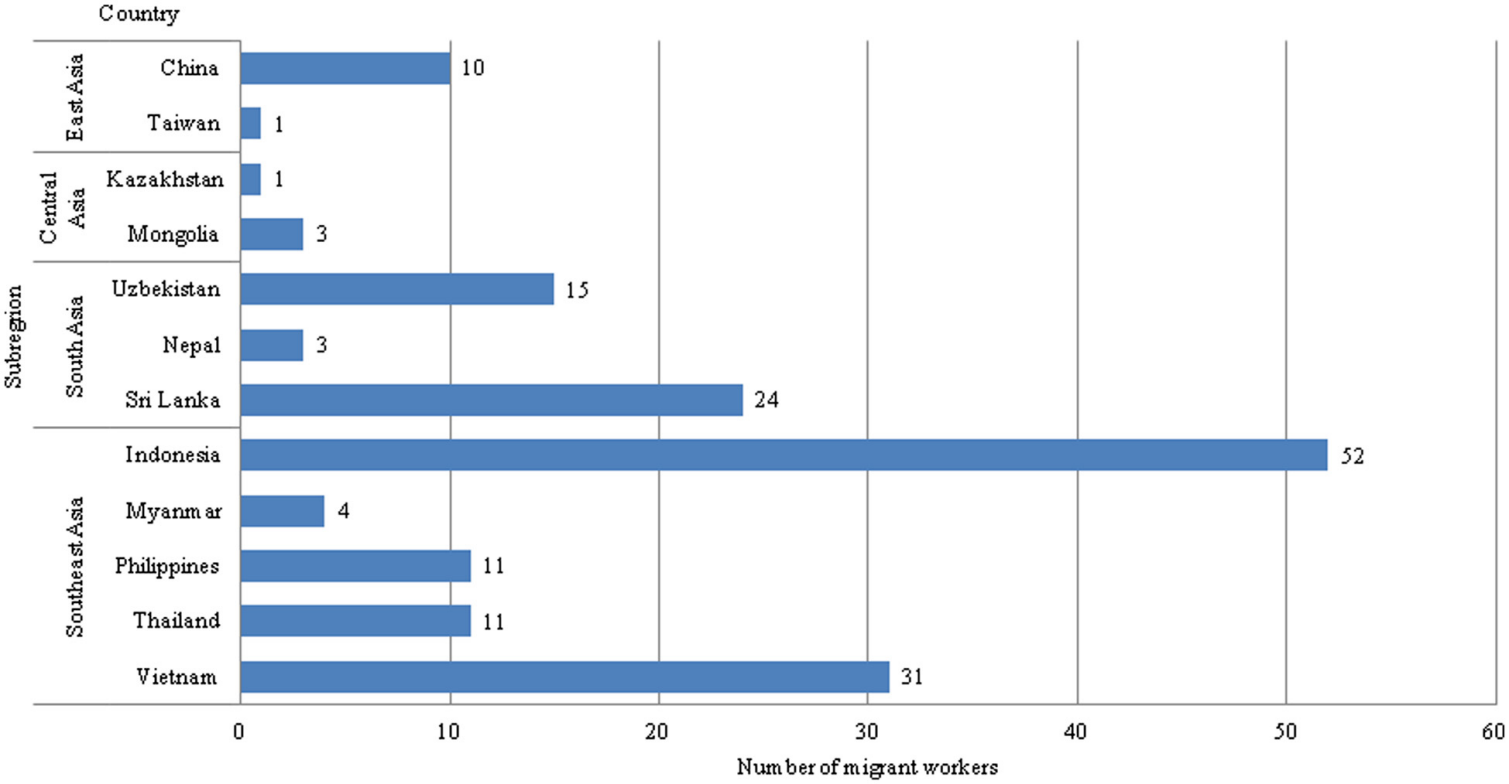
Migrant workers’ country of origin.

Among the 166 migrant workers grouped by region of origin, the group of Southeast Asians was the largest, with 109 subjects. The smallest group was that of East Asians with 11 subjects. Among individual countries, 55 subjects were from Indonesia; on the other hand, there was only one subject from Kazakhstan and one from Taiwan. Most of the subjects, 476 Korean workers (95.6%) and 165 migrant workers (99.4%), were working in the manufacturing sector. The mean age of the migrant workers was 29.0 ± 5.3 years, and the mean age of the Korean workers was 29.3 ± 5.3 years. The highest mean age group was that of the East Asians, with the mean age of 33.9 ± 4.8 years. The lowest mean age group was that of the Southeast Asians, with the mean age of 28.1 ± 5.3 years. The Koreans were significantly taller and heavier than the migrants. The proportion of never smokers was 36.7% in Korean workers and 53% in migrant workers (Figure 1, Table 2).

**Table 2:** **Characteristics of subjects**

**Variables**	**Korean (n = 498)**	**Migrant workers**					***p*** **value** ^*^
		**East Asian (n = 11)**	**Southeast Asian (n = 109)**	**Central Asian (n = 19)**	**South Asian (n = 27)**	**All migrants (n = 166)**	
Age (years)	29.3 ± 5.3	33.9 ± 4.8	28.1 ± 5.3	30.63 ± 5.1	29.3 ± 4.8	29.0 ± 5.3	0.487†
Height (cm)	172.6 ± 5.8	166.9 ± 4.8	165.0 ± 5.4	171.2 ± 5.7	167.9 ± 6.5	166.3 ± 5.9*	<0.001†
Weight (kg)	72.4 ± 11.6	64.2 ± 7.2	62.0 ± 9.1	74.2 ± 14.3	65.4 ± 9.8	64.1 ± 10.5*	<0.001†
BMI (kg/m^2^)	24.3 ± 3.4	23.3 ± 2.6	22.7 ± 2.6	25.1 ± 3.9	23.1 ± 2.7	23.1 ± 2.9*	<0.001†
Smoking history							
Never smokers	183 (36.7)	5 (45.5)	63 (57.8)	8 (42.1)	12 (44.4)	88 (53.0)	0.001‡
Ex-smokers	56 (11.2)	2 (18.2)	5 (4.6)	1 (5.3)	3 (11.1)	11 (6.6)	
Current smokers	259 (52.0)	4 (36.4)	41 (37.6)	10 (52.6)	12 (44.4)	67 (40.4)	
Working duration							
<3 years	282 (56.6)	9 (81.8)	78 (71.6)	19 (100)	22 (81.5)	128 (77.1)	<0.001‡
≥3 years	216 (43.4)	2 (18.2)	31 (28.4)	0	5 (18.5)	38 (22.9)	

Unit: mean ± standard deviation, number (%).

*Differences between Korean and all migrant workers.

†Calculated using *t*-test.

‡Calculated using *χ* ^2^ test.

BMI; Body mass index.

### Comparing the results of applying NHANES III and ethnicity-specific reference equations to the never-smoker group

In all the migrant groups except the Central Asian group, the FEV_1_% and FVC% values calculated using the ethnicity-specific equations were significantly higher than those calculated using the NHANES III reference set. The value of the FEV_1_% of the Central Asian group calculated using the NHANES III reference set and that calculated using the corresponding ethnicity-specific set did not differ significantly. Some differences between the spirometric measurements calculated using both the equations were more than 20%; these are the differences in FVC% in the Southeast Asian group and in FEV_1_% in the South Asian group. The FEV_1_% and FVC% values calculated using the NHANES III reference set were higher for Korean workers than for all the migrant workers in aggregate (*p* = 0.000 for FEV_1_%, *p* = 0.000 for FVC%). In contrast, the result of the FEV_1_% and FVC% calculated using the ethnicity-specific equations for Korean workers were lower than those for all the migrant workers (*p* = 0.015 for FEV_1_%, *p* = 0.000 for FVC%) (Tables 3 and 4).

**Table 3:** **Comparing FEV**_**1**_ **% of never smokers calculated using NHANES III versus ethnicity-specific equations**

	**Korean (n = 183)**	**All migrants (n = 88)**	**East Asian (n = 5)**	**Southeast Asian (n = 63)**	**Central Asian (n = 8)**	**South Asian (n = 12)**
NHANES III	93.59 ± 9.32	85.33 ± 10.90	89.84 ± 5.75	86.07 ± 10.22	94.38 ± 8.67	73.51 ± 8.22
Ethnicity-specific	99.26 ± 10.06†	103.04 ± 12.48	102.1 ± 6.94†	105.27 ± 12.76†	94.03 ± 8.65†	97.62 ± 11.36†
Difference (95% CI)	5.67 (5.43–5.90)^*^	17.70 (16.25–19.15)^*^	12.27 (10.70–13.84)^*^	19.20 (18.45–19.96)^*^	−0.35 (−1.16–0.47)	24.11 (22.04–26.18)^*^

* *p* value < 0.001 according to paired *t*-test.

† Calculated using the relevant ethnicity-specific equation.

FEV; Forced expiratory volume, NHANES; National Health and Nutrition Examination Survey.

**Table 4:** **Comparing FVC% of never smokers calculated using NHANES III versus ethnicity-specific equations**

	**Korean (n = 183)**	**All migrants (n = 88)**	**East Asian (n = 5)**	**Southeast Asian (n = 63)**	**Central Asian (n = 8)**	**South Asian (n = 12)**
NHANES III	92.01 ± 9.33	84.12 ± 11.60	88.84 ± 5.00	84.58 ± 10.52	97.32 ± 7.77	70.97 ± 8.19
Ethnicity-specific	99.68 ± 10.25†	108.63 ± 16.45	104.84 ± 6.59†	114.02 ± 14.87†	100.48 ± 8.32†	87.38 ± 10.98†
Difference (95% CI)	7.67 (7.47–7.88)^*^	24.51 (22.47–26.55)^*^	16.00 (13.97–18.02)^*^	29.44 (28.17–30.71)^*^	3.16 (2.16–4.17)^*^	16.40 (14.21–18.60)^*^

*p value < 0.001 according to paired *t*-test.

† Calculated using the relevant ethnicity-specific equation.

FVC; Forced vital capacity, NHANES; National Health and Nutrition Examination Survey.

In the evaluation of the normal or abnormal FEV_1_ and FVC, the concordance coefficient between applying NHANES III and applying ethnicity-specific equations in the case of the never-smoker Korean workers was 0.378 and 0.433, respectively, which implies moderate agreement. In the case of the never-smoker migrant workers, the concordance coefficient between applying NHANES III and applying ethnicity-specific equations to evaluate the FEV_1_ and FVC was 0.095 and 0.159, respectively, which implies poor agreement. In the evaluation of the abnormal ratio of FEV_1_ and FVC, the abnormal ratio obtained by applying NHANES III was greater than that obtained by applying the ethnicity-specific equations for Korean and all migrants except those of East Asian and Central Asian origins (Tables 5 and 6).

**Table 5:** **Percentage of normal or abnormal FEV**_**1**_ **of never smokers calculated using NHANES III versus ethnicity-specific equations**

		**Korean (n = 183)**	**All migrants (n = 88)**	**East Asia (n = 5)**	**Southeast Asia (n = 63)**	**Central Asia (n = 8)**	**South Asia (n = 12)**
NHANES III	Normal (%)	167 (91.26)	60 (68.18)	5 (100)	44 (69.84)	8 (100)	3 (25.00)
	Abnormal (%)	16 (8.74)	28 (31.82)	0 (0)	19 (30.16)	0 (0)	9 (75.00)
Ethnicity-specific	Normal (%)	179 (97.81)	86 (97.73)	5 (100)	62 (98.41)	8 (100)	11 (91.67)
	Abnormal (%)	4 (2.19)	2 (2.27)	0 (0)	1 (1.59)	0 (0)	1 (8.33)
	Kappa	0.3783	0.095	–†	0.0715	–†	0.0588
	*p* value	0.0005	<0.0001		<0.0001		0.0047

† Kappa not available.

FEV; Forced expiratory volume, NHANES; National Health and Nutrition Examination Survey.

**Table 6:** **Percentage of normal or abnormal FVC of never smokers calculated using NHANES III versus ethnicity-specific equations**

		**Korean (n = 183)**	**All migrants (n = 88)**	**East Asian (n = 5)**	**Southeast Asian (n = 63)**	**Central Asian (n = 8)**	**South Asian (n = 12)**
NHANES III	Normal (%)	163 (89.07)	57 (64.77)	5 (100)	42 (66.67)	8 (100)	2 (16.67)
	Abnormal (%)	20 (10.93)	31 (35.23)	0 (0)	21 (33.33)	0 (0)	10 (83.33)
Ethnicity-specific	Normal (%)	177 (96.72)	84 (95.45)	5 (100)	63 (100)	8 (100)	8 (66.67)
	Abnormal (%)	6 (3.28)	4 (4.55)	0 (0)	0 (0)	0 (0)	4 (33.33)
	Kappa	0.4329	0.1593	–†	–†	–†	0.1818
	*p* value	0.0002	<0.0001				0.0143

†Kappa not available

FVC; Forced vital capacity, NHANES; National Health and Nutrition Examination Survey.

## Discussion

In this study, we made an assumption that Asian migrant workers from the same subregion belonged to the same ethnic classification. Terms such as ethnicity or race are used for the classification of human beings. Ethnicity involves belonging to cultural, sociological aspect of region of origin, and the race classification is based on physical characteristics [27]. However, there are genetic differences between ethnic groups or races; therefore, clinical practices involving racial classification should be carried out carefully [28]. For an accurate spirometric interpretation, it is necessary to know the ethnicity of the subjects tested [14]. Some countries in Asia have a population composed of several ethnic groups. For example, the Singaporean population consists of the major ethnic groups of Chinese, Malays, Indians, and Eurasians [29]. While the country of origin cannot be used to determine race or genetic characteristics with accuracy, it can be used for representing ethnicity.

We could not find reference equations for all the countries of origin of the subjects of this study. Therefore, reference equations developed for people from the same subregions were used. We excluded reference equations developed before 1999 in the selection of equations developed for individuals from a similar spirometric guidelines or socioeconomic status of subjects. Although the ethnicity-specific reference equations applied in this study were developed in Asia, there are some differences among these equations.

Our results demonstrate that in almost all migrant groups, the FEV_1_% and FVC% values calculated using the ethnicity-specific equations and the NHANES III reference set were significantly different. The FEV_1_% and FVC% of Southeast Asians calculated using the ethnicity-specific equation were higher than those calculated using the NHANES III reference set by more than 19%. Further, by comparing the pulmonary function classification of individuals as “normal” or “abnormal” using the ethnicity-specific equations and the commonly used NHANES III set of equations, we found that the proportion of cases with an abnormal FEV_1_ and FVC increased when using the NHANES III set of equations as compared to when using the ethnicity-specific sets. This finding agrees with previous data from other immigrant studies performed in the United States and Israel [30,31]. Further, in a study conducted in India, when the spirometric reference of North Indians was applied to South Indians, the proportion of abnormal values increased [32]. The proportion of abnormal spirometric values of the East Asian and Central Asian groups did not differ significantly between when the NHANES III set of reference equations was applied and when the ethnicity-specific sets were applied. This could be attributed to the limited number of subjects or to the ethnic characteristics of the subjects.

Morris equation [33] and Choi’s equation [34], as well as NHANES III set of reference equation are also widely used as the references for interpretation of spirometry results in Korean. So, although not shown earlier as a result, we compared two reference equations with the relevant ethnicity-specific equations. We found that all the migrant groups except the Central Asian group, the FEV_1_% and FVC % values calculated using the relevant ethnicity-specific equations were significantly higher than those calculated using the Morris equation and Choi’s equation. In the evaluation of the spirometric results of subjects from Southeast Asia or South Asia, the percentage of abnormal FEV_1_ increased when the Morris equation and Choi’s equation were used instead of the relevant ethnicity-specific equations. These results were very similar to compared NHANES III reference set with the relevant ethnicity-specific equations.

Ethnic origin affects pulmonary prediction values. The ERS/GLI 2012 equation provides age, height, sex, and ethnicity-specific reference equations and the LLN for spirometry [12]. However, thus far, no definite valid explanation for differences in the normal lung function parameters attributed to ethnicity has been reported. Nevertheless, the literature suggests that inherited differences, anthropometric differences, environmental and social factors among ethnic groups play a role [35].

In 2012, in Korea, there were 128,274 migrant male workers from Southeast Asia, 47,552 from South Asia, 22,773 from Central Asia, and 6,199 from East Asia. With respect to the country of origin, the largest number of migrant workers (52,084) were from Vietnam, followed by 24,701 from Indonesia, and then, 19,830 from Sri Lanka, 15,116 from Uzbekistan, and 14,619 from Nepal. Similarly, according to the number of migrant workers, our subject groups were from Southeast Asia, South Asia, Central Asia, and East Asia [4]. Pulmonary function of the migrant workers are currently being calculated using the same equation as that is used for Koreans. Therefore, we should pay attention to their normal lung function predictive values.

This study has several limitations and possible confounders. Firstly, all subjects were employees undergoing a routine annual health examinations at a health exam center located in Busan city. This may have caused a selection bias due to the “healthy worker effect”. Further, the material hazardous to the pulmonary system by occupational exposure was not considered. However, the migrants’ working conditions were almost all similar. Another possible limitation is the fact that the selection of subjects was not representative of all migrant workers in Korea. Nevertheless, we think that the results of this study should be typical of spirometry results from migrant workers throughout Korea.

This study showed that the spirometric results of migrant workers from Southeast Asia or South Asia can be calculated as too low when obtained using the same equation as that used for Koreans. Laws requiring regular health check-ups of workers would support the health management of workers exposed to hazardous materials at the workplace. However, an overdiagnosis of the pulmonary dysfunction of migrant workers can influence the assessment of their work fitness and consequently, limit the occupational options of migrant workers. Therefore, an appropriate standard for evaluating the spirometric results of migrant workers should be established.

## Conclusions

The number of migrant workers in Korea is increasing. According to Korean law, employees exposed to pulmonary hazards in the workplace must undergo regular health check-ups including spirometry. This study showed that all the migrant groups except the Central Asian group, the FEV_1_% and FVC% values calculated using the ethnicity-specific equations were significantly higher than those calculated using the NHANES III reference set. And in the evaluation of the spirometric results of subjects from Southeast Asia or South Asia, the percentage of abnormal FEV_1_ or FVC increased when the NHANES III reference set was used instead of the relevant ethnicity-specific equations.
